# Recent Advances in Novel Antiviral Therapies against Human Adenovirus

**DOI:** 10.3390/microorganisms8091284

**Published:** 2020-08-22

**Authors:** Bratati Saha, Robin J. Parks

**Affiliations:** 1 Regenerative Medicine Program, Ottawa Hospital Research Institute, Ottawa, ON K1H 8L6, Canada; bsaha021@uottawa.ca; 2 Department of Biochemistry, Microbiology and Immunology, University of Ottawa, Ottawa, ON K1H 8M5, Canada; 3 Centre for Neuromuscular Disease, University of Ottawa, Ottawa, ON K1H 8M5, Canada; 4 Department of Medicine, The Ottawa Hospital, Ottawa, ON K1H 8L6, Canada

**Keywords:** human adenovirus, antiviral therapy, nucleoside analogues, cardiotonic steroids, corticosteroids, HDAC inhibitors, methyltransferase inhibitors, small molecule screens, vaccines

## Abstract

Human adenovirus (HAdV) is a very common pathogen that typically causes minor disease in most patients. However, the virus can cause significant morbidity and mortality in certain populations, including young children, the elderly, and those with compromised immune systems. Currently, there are no approved therapeutics to treat HAdV infections, and the standard treatment relies on drugs approved to combat other viral infections. Such treatments often show inconsistent efficacy, and therefore, more effective antiviral therapies are necessary. In this review, we discuss recent developments in the search for new chemical and biological anti-HAdV therapeutics, including drugs that are currently undergoing preclinical/clinical testing, and small molecule screens for the identification of novel compounds that abrogate HAdV replication and disease.

## 1. Introduction

Human adenovirus (HAdV) is a non-enveloped DNA virus that mainly causes self-limiting illnesses in most patients, but can lead to severe disease and death in others. HAdV-induced disease can rapidly spread through confined populations such as daycare centers, schools, hospitals, retirement homes and military training venues [1]. HAdV was first isolated from adenoid tissue and determined to be the causative agent in some respiratory infections in the 1950s [2,3]. To date, over 80 HAdV types have been identified, which are grouped into seven species (A–G) [4]. Of these, types 2 and 5 (species C) are the most extensively studied. Investigations on how HAdV proteins interact and modify cellular components have led to the elucidation of many crucial host protein/pathway functions. For example, alternative splicing was first identified in HAdV, and the histone acetyltransferase (HAT) p300 and chaperone EP400 were discovered through their interaction with the HAdV E1A protein [5,6,7,8]. Furthermore, examination of the HAdV infection process and lifecycle have provided novel information on fundamental cellular processes, including DNA replication, cell cycle progression and RNA splicing [9]. Thus, studies on HAdV have led to significant insights in virus biology, infection process, host–pathogen interactions and the manipulation of cellular processes [10,11].

HAdV spreads effectively mainly via oral/nasal secretions (and the fecal route in some cases) and can cause a variety of diseases depending on the type [12,13,14,15,16,17,18,19,20,21,22,23,24]. No approved antiviral therapy currently exists for the treatment of severe HAdV infections [17,18,24,25]. A number of DNA/RNA synthesis-inhibiting antivirals that are approved for the treatment of other viral infections (such as cidofovir, ganciclovir and ribavirin) are currently used off-label to treat severe HAdV infections in the clinic [16,17,18,21,22,25,26]. However, limited efficacy and serious adverse effects have been observed with these drugs in many cases [16,18,21,22,25,27,28]. HAdV infections and current treatment options are further discussed in Section 5. In addition, new strains of HAdV are continually being isolated from patients from around the world, and these emerging strains, which result from zoonotic transmission in some cases [29], may vary in virulence and susceptibility to existing antivirals [4,30,31,32,33]. Consequently, it is necessary to develop additional, more effective anti-HAdV compounds. Over the last decade, many novel compounds (including drugs approved for treatment of other conditions) have been reported to exhibit antiviral activity against HAdV in vitro and in vivo. This review will summarize the current treatment options for HAdV-induced disease and discuss the recent advances in the discovery of antiviral compounds against HAdV in pre-clinical studies. Although this article was not prepared as a formal systematic review, an attempt was made to include the majority of reports available to date in peer-reviewed journals on novel compounds with anti-HAdV activity.

## 2. HAdV Biology: The Viral Genome

**Figure 1:**
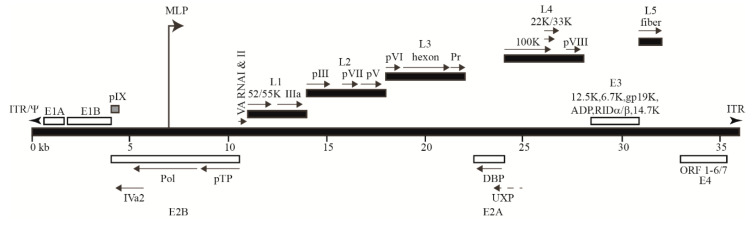
Schematic representation of the Human adenovirus (HAdV)-2 genome (36 kb). Relative locations of the early (E1–E4, white boxes) and late (L1–L5, black boxes) coding regions and the major late promoter (MLP) are shown. Transcription units depicted above the genome bar are transcribed in a rightward orientation, whereas those below are transcribed in a leftward orientation. Both ends of the linear viral genome contain inverted terminal repeats (ITRs), which serve as the origins of replication. The genome packaging sequence (**Ψ**) is located adjacent to the left ITR. This diagram was generated primarily based on the sequence information provided by Zhao et al. [36].

HAdV is a nuclear virus with most of its lifecycle, including viral gene transcription, genome replication and progeny virion formation, occurring within the nucleus of the infected cell [10]. The size of the double-stranded viral genome varies based on type (26–45 kb) and encodes ~40 proteins within its “early” and “late” transcription units (Figure 1), which are expressed before and after viral DNA replication, respectively [9,34]. The information provided below on virus biology and lifecycle mainly pertain to HAdV-2 and HAdV-5, which are ~95% identical at the nucleotide level [35]. However, much of this information is relevant to other HAdV types.

### 2.1. The Early Genes

As with all other viruses, HAdV relies on host cell machinery for almost all steps of the virus lifecycle. The HAdV early regions (E1A, E1B, E2, E3 and E4) are the first viral proteins to be expressed during infection [37,38,39]. The early region 1A (E1A) proteins are crucial for efficient viral gene expression and replication as they stimulate the expression of all other viral genes [40]. Differential splicing of the primary E1A transcript produces five different transcripts, of which the 13S and 12S mRNAs are the most abundant in early infection and the 9S is the major species during late infection [41]. However, all five forms of the E1A protein can be detected in vitro during late infection. The major forms, the 289 residue (R) and the 243R proteins (resulting from the 13S and 12S mRNAs, respectively), interact with a variety of cellular proteins including cell cycle regulators, epigenetic regulators and transcription factors [5,6,42,43,44,45,46]. These interactions, among other functions, serve to enhance viral gene expression, alter the cellular gene expression profile, modulate the function of host proteins, and induce mitogenic activity within the host cell to promote a productive infection [9,37,47,48,49]. The 55 kDa protein product from the E1B region has been implicated in the ubiquitination of cellular proteins (e.g., p53), the inactivation of cellular DNA damage response and the regulation of viral mRNA export [50,51].

The early region 2 (E2) consists of two transcriptional units, E2A and E2B, which have separate polyadenylation sites and encode the proteins required for viral DNA replication [52,53]. E2A codes for the DNA-binding-protein (DBP), while E2B codes for the precursor terminal protein (pTP) and the viral DNA polymerase (Pol). In the protein-primed HAdV DNA replication process, pTP-bound viral DNA serves as the template. Elongation by Pol occurs through a strand-displacement process, during which, DBP forms multimers on the displaced single-stranded DNA (ssDNA) [54,55]. Although the main function of DBP is to drive template unwinding, it may also protect the ssDNA by preventing immune and DNA damage responses that can be elicited in the cell against naked DNA from foreign species [54,56,57]. During virion maturation in the late phases of infection, pTP is cleaved by the HAdV-encoded protease, resulting in the mature terminal protein (TP) that remains associated with each 5’ end of the newly-replicated DNA within progeny virions [58,59].

Proteins from the E3 region mostly have immunomodulatory functions within infected host cells [38,60,61]. The E3 region also encodes the HAdV death protein (ADP), which increases the efficiency of cell lysis and virus progeny release [62]. Since the E3 proteins are not essential for HAdV replication in vitro, this region is frequently deleted, and often replaced with foreign expression cassettes for generating viral vectors with therapeutic transgenes or fluorescent protein genes to monitor virus entry/localization. Lastly, the E4 proteins alter cellular protein stability/activity (e.g., E4orf3 and E4orf6 assist the E1B-55 kDa protein in neutralizing p53 activity), as well as signaling pathways to regulate viral RNA splicing and prevent the virus-induced activation of the DNA damage response [39,50,63,64].

In summary, the early proteins have diverse functions in the infected cell, which ultimately allow HAdV to hijack cellular pathways to promote viral gene expression and make the cellular environment conducive to replication. Although antivirals specifically targeting early proteins have not yet been discovered/designed, any drug that interferes with the expression or function of these proteins, especially those from the crucial E1 and E2 regions (as well as the late regions discussed below), could impair efficient virus replication.

### 2.2. The Late Genes

**Figure 2:**
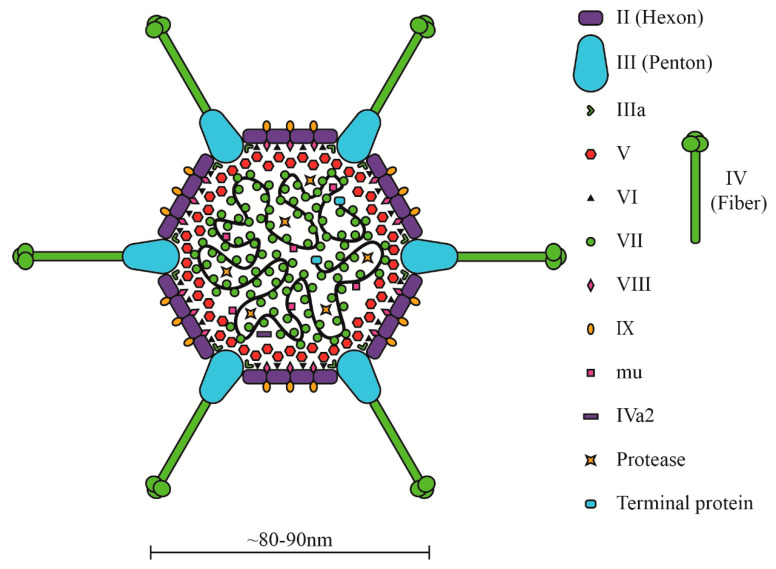
Structure of the HAdV virion. The majority of capsid proteins (e.g., penton, hexon, fiber) and core proteins (e.g., pVII, terminal protein, mu) associating with the viral DNA (black line) are shown. This illustration was obtained from Saha et al., Figure 2 [69], and is used herein under the Creative Commons CC BY 4.0 license.

The HAdV genome encodes five late transcription units, L1–L5 (Figure 1), which code for virus structural/capsid (e.g., penton, hexon, fiber) and core (e.g., protein VII, protease) proteins (Figure 2) [65]. The regulation of late gene expression is under the control of a common major late promoter (MLP), which is fully activated following the onset of viral DNA replication in late infection [66]. A single transcript containing five different polyadenylation sites is first produced, and five groups of transcripts are then generated by alternative splicing [36,67]. Another late promoter within the L4 region was more recently identified, which allows for low-level expression of the L4-22K and L4-33K proteins that are involved in inducing the full activation of the MLP [68]. The late structural proteins are further discussed in Section 3.

In addition to the major early and late proteins discussed above, several other small products are produced from the HAdV genome during intermediate (protein IX or pIX, IVa2) and late (virus-associated (VA) RNA I, VA RNA II and the U exon protein (UXP)) infection (Figure 1). pIX, a structural protein expressed after the initiation of early gene expression and before late gene expression, plays a role in virion stability [70]. VA RNA I and II are double-stranded RNA molecules that promote viral protein synthesis while impairing interferon-mediated antiviral responses and cellular micro-RNA processing [71,72,73]. UXP has been suggested to play a role in virus DNA replication or RNA transcription [74,75].

## 3. HAdV Biology: The Virion

The general structure of mature, infectious virions is relatively conserved among HAdV species. The non-enveloped, icosahedral capsid mainly consists of hexon, penton and fiber proteins (Figure 2) [76,77,78,79]. The latter is a long protein that emerges from each vertex of the icosahedron, significantly increasing the overall capsid diameter (Figure 2). Fiber is crucial for HAdV-5 attachment and entry into host cells. Inside the virion, the HAdV DNA is bound to basic proteins VII, V and mu (Figure 2) [80]. Both protein VII (pVII) and mu condense and compact the DNA within the virion [81,82]. Protein V surrounds the pVII-DNA complex and is believed to act as a bridge to link the core to the capsid [80,83,84,85]. Five other minor proteins (IIIa, IVa2, VI, VIII and IX; see Figure 2) either assist with stabilizing the capsid, connecting the capsid to the nucleoprotein core or packaging the viral genome [86,87].

## 4. Infection and Virus Lifecycle

Cell surface receptors used by HAdV can vary based on the species, but the coxsackie and adenovirus receptors (CAR) are primarily used by species C, including HAdV-2 and HAdV-5 [88]. The binding of the fiber protein to the CAR, which is expressed by most cell types, initiates virus entry into host cells [88,89]. Subsequently, the interaction of an arginine–glycine–aspartic acid (RGD) motif in the penton protein with cell membrane-associated αvβ3 or αvβ5 integrins induces virus internalization by receptor-mediated endocytosis into clathrin-coated vesicles [90,91,92,93]. These vesicles eventually form endosomes, from which the virus escapes by lysing the early endosomal membrane [91]. The virions then migrate to the cell nucleus using the cellular microtubule network [94,95]. The capsid proteins are removed during transport, such that only the pVII-wrapped and TP-bound viral DNA enter through the nuclear pore complex [96,97,98].

During the early phase of infection, pVII is replaced by histones in the cell nucleus, which arrange the DNA into a nucleosome-like structure similar to cellular chromatin [99,100,101,102]. The nucleosome density on the viral genome is significantly reduced during late infection [101,103,104]. During later stages of DNA replication and nascent genome packaging into newly formed capsids, the viral genome copies must sequentially associate with DBP and pre-pVII, respectively [105]. Thus, the viral and cellular proteins that associate with the HAdV genome are dynamically regulated throughout infection.

Viral DNA replication occurs soon after early gene expression, which allows the full activation of the MLP and, subsequently, the expression of late genes [66]. Following their synthesis in the host cell cytoplasm, the structural proteins (e.g., fiber, penton, hexon) are transported into the nucleus for the assembly of progeny virions [79,106]. HAdV capsids likely form first, followed by the packaging of the DNA into the empty capsids which requires the viral packaging element (**Ψ**), IVa2 and other viral proteins [65,107]. Finally, the death of infected cells from virus-induced cytopathic effects (CPE) and the release of progeny virions conclude the HAdV lifecycle. At these later stages of infection, the virus inhibits gene expression and protein synthesis in the infected host cell, which essentially serves as a virus production factory until death due to CPE [108].

## 5. HAdV-Induced Disease and Current Treatment Options

HAdV transmission mainly occurs via aerosol droplets and the fecal–oral route [22]. The virus causes minor, self-limiting illnesses in most immunocompetent patients. Such mild infections are highly underreported, leading to an underestimation of HAdV infection and prevalence in the population [109]. In 2014, a voluntary reporting system called the National Adenovirus Type Reporting System (NATRS) was created by the US Centers for Disease Control and Prevention (CDC) to collect information on HAdV types circulating in the United States [19].

### 5.1. HAdV Infections

HAdV has a remarkable capacity to spread as an infected patient can contract the disease from only five virus particles [12]. To place this in perspective, sputum/oral secretions from an infected adult and blood from patients with disseminated infection can contain between 10^6^ and 10^7^ virus particles per milliliter [13]. Although symptoms in most infected patients are mild and resolve within a few days without intervention, HAdV can cause severe disease, including hemorrhagic cystitis, hepatitis, myocarditis and neurologic complications, leading to respiratory/multiorgan failure and even death in pediatric, geriatric and immunocompromised patients (e.g., stem cell or organ transplant recipients) [14,15,16,17,18,19,20,21,22]. In fact, HAdV-induced disease can be fatal in up to 80% of neonates with systemic infection and in ~40% of children with infection-related neurologic complications [18,110]. In a recent HAdV-7 outbreak at the Wanaque Center for Nursing and Rehabilitation in New Jersey, ~35 people were infected, including 23 children. Eleven of the 23 pediatric patients succumbed to the infection [111]. Several cases of immunocompetent patients succumbing to HAdV-induced respiratory failure have also been documented [112,113].

The tissues/organs affected by HAdV-induced disease and the severity varies based on the virus type involved [19,23,24]. Of the seven species of HAdV, species B, C and E are most frequently associated with upper respiratory tract infections: species C (types 1, 2, 5) mainly causes mild infections in young children, while species B (types 3, 7, 14, 21) and E (type 4) cause more severe infections in both children and adults [19,23,109]. Although HAdV accounts for only ~5% of all childhood respiratory tract infections, it can lead to bronchitis or pneumonia, requiring hospitalization in many cases [114,115]. Species D is associated with conjunctivitis, and species A and F are responsible for causing infections of the gastrointestinal (GI) tract [116]. GI-associated HAdV types are reportedly a major causative agent of disease in children below the age of 5 in low- and middle-income countries [117]. Overall, HAdV infection can be severe and fatal in some populations, and is a significant burden to society due to the loss of work hours and associated medical expenses [115].

### 5.2. Current Antiviral Therapies

No approved antiviral therapy currently exists for the treatment of severe HAdV infections [17,18,24,25]. Cidofovir, a cytosine analogue that interferes with viral DNA synthesis which is an approved antiviral agent against cytomegalovirus (CMV), is considered to be the standard treatment in most cases [16,18,21,22,25,26]. Several other DNA/RNA synthesis inhibitors used as antivirals in other viral infections (e.g., ganciclovir, ribavirin) are also used to treat HAdV infections in the clinic [16,17,18,21,22]. However, these drugs are often associated with serious adverse effects (such as nephrotoxicity) [16,22,25,27], limited efficacy, and poor disease prognosis in systemic HAdV infections in immunocompromised patients [18,21,28]. For example, ganciclovir, a common antiviral against CMV and herpes simplex virus type 1 (HSV-1), is less effective in HAdV infections, as the drug must first undergo phosphorylation to an active form and unlike CMV and HSV-1, HAdV does not encode a viral kinase to facilitate this modification in infected cells [118]. Brincidofovir, a phospholipid conjugate of cidofovir, is currently in clinical trials and has received FDA Fast Track status for treatment of HAdV-induced diseases [119,120]. This drug has several advantages over cidofovir, including oral delivery and increased cellular uptake. However, brincidofovir provides only modest benefits and can cause GI toxicity in some patients [25,120]. Immunotherapies assessing virus-specific T-cells are also undergoing clinical trials [25,121]. Thus, considering the limited and relatively ineffective options currently available, more effective anti-HAdV therapies are necessary to combat severe HAdV infections [26,28].

### 5.3. Anti-HAdV Vaccines

A live, oral vaccine against types 4 and 7 was used by the U.S. military from 1971 to 1997 to prevent HAdV disease in new recruits [19]. The vaccine (now mandatory) was later reintroduced in 2011 due to a substantial increase in HAdV infections among military personnel during the intervening period of non-vaccination [1]. This vaccine is very safe and effective, and it led to a 300-fold decrease in disease burden within the first two years of reintroduction [22]. However, the vaccine is not available to civilians.

## 6. Discovery of New Antiviral Therapies

The need for novel anti-HAdV therapies has been highlighted by many clinical and case studies, and several research groups have discovered novel compounds that exhibit considerable activity against HAdV in cell culture and in animal models (Table 1). Many of these compounds are FDA-approved therapeutics for other illnesses, such as antivirals targeting other DNA/RNA viruses and histone deacetylase (HDAC) inhibitors that are used as chemotherapy agents for cancer treatment.

### 6.1. Nucleoside/Nucleotide Analogues

Since cidofovir is often used as a treatment option for HAdV infections in the clinic, various derivatives of the drug and other nucleoside/nucleotide analogues have been evaluated for their activity against HAdV. Many of these compounds were either comparable to or more effective in inhibiting HAdV than the parental drug in vitro and in vivo [118,122,125,128]. In a study by Naesens et al., acyclic nucleoside phosphonate analogues of cidofovir were the most effective in reducing HAdV-2 DNA replication and infectious particle yield from infected cells [118]. Several ether lipid-ester prodrugs of cidofovir, as well as the acyclic nucleoside phosphonates, were even more active than cidofovir against several HAdV types in a separate, independent study [125].

Among other nucleoside/nucleotide analogues tested against HAdV, 2′,3′-dideoxycytidine, also known as zalcitabine, an FDA-approved anti-retroviral for the treatment of HIV infections, exhibited considerable anti-HAdV activity within a clinically achievable concentration range in several in vitro investigations [118,122,123]. Another anti-HIV agent, stavudine (a thymidine analogue) was also found to be potent against HAdV [124]. Furthermore, we observed that gemcitabine, a cytidine analogue and a chemotherapeutic agent, abrogates gene expression and the replication of HAdV-4, HAdV-5 and HAdV-7 [126], while cytarabine led to modest effects on HAdV-5 gene expression and yield [127]. These compounds may prove to be useful in cases where cidofovir fails to improve disease outcome, but thorough assessment of their efficacy and safety in animal models is required prior to clinical testing.

### 6.2. HDAC Inhibitors and Inhibitors of Other Epigenetic Regulatory Proteins

The pVII-bound viral DNA is not permissive for efficient transcription or replication [81]. Soon after reaching the nucleus, the HAdV genome associates with histones (with a preference towards the H3 variant H3.3), and adopts a repeating nucleosome-like structure similar to the host DNA [99,100,101,103,104,141]. This template facilitates the expression of the viral early genes that are crucial for establishing a successful infection.

The presence of H3 acetylation at highly active HAdV-5 promoters was first observed almost a decade ago [99]. Since then, a few research groups have reported the presence of H3 (at residues K9, K14, K18 and/or K27) and H4 acetylation at many of the early promoters and at the MLP [142,143,144]. Although the level of acetylation was found to be differentially regulated depending on the genome region and infection time, acetylation generally served to recruit TATA-binding protein/RNA polymerase and activate transcription from the early promoters. Overall, the data suggests that the HAdV genome likely has a complex temporal/regional requirement of histone acetylation for efficient promoter function. These observations also suggest that the cellular epigenetic modifiers involved in transitioning the HAdV genome to a transcriptionally-active nucleoprotein structure, and in regulating viral gene expression, may serve as valid targets for therapeutic intervention. Indeed, such an approach has been effective against HSV, where the inhibition of histone demethylases prevented virus lytic replication and reactivation from latency [145,146,147,148]. In the case of HAdV, valproic acid-mediated inhibition of HDAC activity prevented HAdV-5 replication and spread in cell culture [129]. Consistent with this report, we showed that the treatment of infected cells with various HDAC inhibitors suppressed virus-encoded gene expression [130]. Furthermore, the treatment of infected cells with the pan-HDAC inhibitor vorinostat (or SAHA) had a negative impact on multiple other stages of the virus lifecycle including early/late gene transcription, viral DNA replication and the production of progeny virions. Vorinostat also had efficacy against more virulent and clinically relevant types 4 and 7. This effect was primarily due to the loss of class I HDAC activity, mainly HDAC2. However, in a recent report, HDAC inhibitors were shown to promote latent HAdV reactivation in tonsillectomy samples [149], perhaps suggesting differential reliance on HDACs in lytic versus latent infections.

In addition to HDAC inhibitors, several methyltransferase inhibitors have also shown efficacy against HAdV. For example, Arbuckle et al. reported that the inhibitors of methyltransferases EZH2 and EZH1 (which deposit the repressive histone H3K27 tri-methylation mark), and histone H3K9 demethylase LSD1 successfully attenuated HAdV gene expression [131,148]. Interestingly, HDAC inhibitors can suppress LSD1 activity [150], which may contribute to their anti-HAdV properties. We also recently discovered that chaetocin (primarily inhibits the H3K9 methyltransferases SUV39H1 and G9a/GLP) and lestaurtinib (inhibits JAK2 and PRK1, which can phosphorylate residues on H3 in addition to other cellular targets) are potent inhibitors of viral gene expression and DNA replication that can lead to a ~100-fold reduction in the virus yield in cell culture [126]. Further studies are required to determine whether these latter compounds exert their effect through epigenetic means or through some other mechanism.

Interestingly, the HDAC/methyltransferase inhibitors with anti-HAdV activity target cellular epigenetic regulatory proteins that typically confer repressive post-translational modifications (PTMs). If these proteins were naturally acting to suppress viral gene expression through these repressive PTMs (either on the viral genome-associated histones or the genome itself), treatment with their inhibitors would be expected to enhance virus gene expression. However, based on the data discussed above, the modifications performed by these regulatory proteins may modulate host gene expression/protein activities such that efficient virus replication is promoted. During a productive infection, HAdV proteins may engage these epigenetic regulators to modulate cellular gene expression and allow virus replication to occur efficiently. Such a mechanism has already been described for HAdV: the HAdV E1A protein interacts with and redirects p300/CBP histone acetyltransferase to epigenetically reprogram cellular chromatin, thereby conferring a global change in cellular gene expression to promote an environment conducive for virus replication [151,152]. Based on this scenario, the inhibitors of these epigenetic regulators would reduce virus replication. Although the mechanisms by which the inhibition of HDACs/methyltransferases affect HAdV replication remain to be elucidated, they appear to be valid therapeutic targets to limit infection and virus spread.

### 6.3. Steroid-Based Compounds

The anti-HAdV activity of steroid-based compounds has been reported by a number of research groups [127,132]. Changes in the localization of SR proteins, which are involved in cellular RNA splicing, by cardiotonic steroids digoxin and digitoxin was shown to affect HAdV-5 early RNA processing. Consequently, the relative levels of the spliced E1A transcripts (and thus, E1A protein isoforms) were altered, leading to drastic reductions in virus replication and yield at nanomolar concentrations [132]. The treatment of infected cells with digoxin, digitoxigenin and lanatoside C led to very similar outcomes for virus replication in one of our recent investigations, where these three cardiotonic steroids were the top hits in a small molecule screen we conducted to identify novel HAdV inhibitors (see Section 6.6) [127]. These cardiotonic steroids reduced the late gene expression from HAdV-4 and HAdV-7 in our study, and the recovery of HAdV-31, HAdV-35 and a species D clinical isolate in a previous study, suggesting broad-spectrum activity against multiple types of HAdV [127,132]. Cardiotonic steroids can inhibit other DNA viruses, such as CMV and HSV as well [153,154,155]. In these cases, impacts on gene expression, protein translation and virus release were implicated as the mechanism of inhibition, which may contribute to the efficacy of these compounds against HAdV. Along with the cardiotonic steroids, corticosteroids (dexamethasone, flunisolide, flumethasone, diflorasone and flurandrenolide) were also identified as positive hits in our screen. Dexamethasone and flunisolide modestly affected late protein levels in subsequent validation experiments, resulting in a lower virus yield [127].

In a study by Marrugal-Lorenzo et al., mifepristone, a commercially available synthetic steroid, reduced HAdV replication at micromolar concentrations in cells and in mice [133]. The compound appeared to interfere with virus entry into the nucleus as well as the replication of the viral genome. Natural steroid hormones epiandrosterone, dehydroepiandrosterone and several derivative compounds were reported to reduce HAdV replication by impacting protein production, while entry/adsorption was unaffected [134]. None of the steroid-based compounds discussed here have yet been tested in appropriate animal models (such as the cotton rat or the Syrian hamster) of HAdV infection or in clinical studies [156].

### 6.4. Other Compounds with Anti-HAdV Activity

In addition to various nucleic acid synthesis inhibitors, protease inhibitors have been evaluated against HAdV. Nelfinavir mesylate—the active ingredient of Viracept, which is an FDA-approved anti-HIV drug that functions by inhibiting the HIV aspartyl protease—was shown to prevent lytic egress and the cell-to-cell transmission of several HAdV types [139]. HAdV protease-specific inhibitors were identified by McGrath et al. via structure-based drug design in silico [140].

Among other categories of small molecules with antiviral activity against HAdV, piperazine-derived compounds have shown promise in several investigations [135,136]. A trisubstituted piperazin-2-one derivative was identified in a small molecule screen (further discussed in Section 6.6), and inhibited viral DNA replication at low micromolar concentrations, with minimal cytotoxicity [135]. Tazarotene, a selective agonist of the retinoic acid receptor β (RARβ), was investigated in another study as a HAdV replication inhibitor after the transcriptomic analysis of infected A549 cells revealed that the virus induced the downregulation of RARβ mRNA [137]. Very recently, ivermectin was found to inhibit gene transcription, protein expression, genome replication, and viral progeny production of HAdV-5 and the more clinically relevant HAdV-3 [138]. Ivermectin disrupted the binding of the E1A protein to importin-α, preventing E1A import into the nucleus. Other miscellaneous and broad-spectrum antiviral compounds with anti-HAdV activity (e.g., cycloferon, lactoferrin, camptothecin) have been previously described [124,157]. The identification of such compounds and the elucidation of their mechanism of inhibition may eventually lead to the discovery of more effective therapeutic agents to treat HAdV-induced disease.

### 6.5. Vaccines and Biological Antivirals against HAdV

As discussed in Section 5.3, due to the re-emergence of HAdV-induced disease in military training venues, the United States Department of Defense redeveloped a live oral vaccine for HAdV-4 and HAdV-7 [1]. In phase III clinical trials, this vaccine was ~95% effective at inducing antibody production against the two types [158]. The development of novel vaccines against other HAdV types is a relatively unexplored field. To date, only a limited number of inactivated and recombinant multivalent vaccines against clinically relevant HAdV types have been investigated. The majority of these vaccines contain hexon as the target antigen, because host-neutralizing antibodies are primarily directed against this protein [114]. Fiber, penton and other capsid proteins are also immunogenic [159]. Liu et al. showed that a trivalent vaccine against three types (3, 7 and 55, all responsible for acute respiratory disease) induced the production of antibodies, reduced virus load and improved lung pathology in a mouse model of infection [160]. Other types of non-small molecule antivirals explored against HAdV include RNA interference (e.g., siRNAs and microRNAs targeting viral mRNAs) in non-clinical research, and intravenous immunoglobulin therapy and T-cell immunotherapy in clinical settings [124,157,161].

### 6.6. Small Molecule Library Screens

Efficient HAdV replication and growth rely on the ability of the virus to take advantage of endogenous protein activities and pathway functions in the host cell [5,11,38,39,51,151,153,162,163,164]. Thus, in theory, virus replication can be inhibited by compounds that target various cellular proteins required at different stages of the virus lifecycle. Small molecule screens can enable the discovery of such antiviral compounds with previously unexploited virus/host targets and diverse mechanisms of action.

The high-throughput screening of small molecules is a widely used, cost-effective drug-discovery approach that has led to the identification of novel therapeutics or the repurposing of existing approved drugs to combat a variety of viral infections [165,166,167,168]. However, the majority of new HAdV inhibitors have been identified through investigations on a single or a handful of select compounds [132,133,136,137]. To date, very few drug library screens have been performed to find novel anti-HAdV compounds [126,127,135,169,170,171]. Many of these screens were conducted using non-replicating HAdV vectors that expressed the green fluorescent protein (GFP) from a heterologous promoter, which may not accurately mimic wild-type virus gene expression and replication [135,171]. A fluorescence-based high-throughput screen was designed by Duffy et al. to specifically identify small molecules that inhibited the coagulation factor X (FX)-mediated uptake of HAdV-5 vectors by hepatocytes [170]. In total, 10,240 molecules were screened, and three positive hits were found to be effective in blocking the intracellular transport of the virus at low micromolar concentrations. Since the design of this screen was aimed at discovering HAdV transduction inhibitors, compounds impairing other stages of the virus lifecycle were not identified.

More recently, a screening platform using luminescence-based detection of cytotoxicity and CPE was developed by Hartline et al. This approach, where the antiviral activity of a given compound is quantified by analyzing the difference in drug-induced cytotoxicity and virus-induced CPE, allowed for broad-spectrum screening for antiviral compounds against various DNA viruses including HAdV [169]. The investigators identified two compounds with previously unreported anti-HAdV activity: FCV and 4-thio-IDU. Although this assay offers the advantage of selecting antiviral compounds at a desirable concentration with low cytotoxicity, it relied on more indirect measurements of viral gene expression/replication, which may have limited the ability of the screen to detect compounds with modest effects on virus replication.

To more effectively screen for small molecules that directly impact HAdV replication and gene expression, we designed a wild-type-like HAdV-5 reporter construct in which the red fluorescent protein (RFP) coding sequence was placed under the regulation of the MLP [130]. As RFP production from this reporter construct occurs only following active virus replication and is concomitant with HAdV late gene expression, fluorescence can be used to monitor the effects of compounds on viral gene transcription, protein production/stability and replication in cell culture [130]. We used this platform to screen ~1200 FDA-approved compounds from the Prestwick Chemical Library [127], and ~150 compounds from the Cayman Epigenetics Screening Library [126]. Positive hits from these screens consisted of diverse classes of compounds, such as cardiotonic steroids/cardiac glycosides, corticosteroids, chemotherapeutic agents, HDAC inhibitors and other modulators of epigenetic regulatory proteins (see Section 6.1, Section 6.2 and Section 6.3). This screening platform offers sufficient sensitivity to detect compounds that induce small or transient reductions in viral gene expression/replication, as well as those with more robust antiviral activity. More importantly, the procedure can be easily adapted for screening much larger drug libraries in a high-throughput manner to facilitate drug discovery and repurposing for ameliorating HAdV-induced disease.

## 7. Concluding Remarks

The U.S. Department of Defense has detailed data on the human cost of HAdV-induced disease [1,22,172]. After 28 years (1971–1999) of active vaccination of all recruits, the manufacturer stopped making the HAdV vaccine, and cases of HAdV-associated febrile respiratory illness rose dramatically (~15,000 patients per year between 1999 and 2004). Up to 30% of all new recruits sought treatment during periods of high transmission. While this may be an extreme example due to the close proximity in which military recruits live and work, it does illustrate the impact HAdV can have in certain populations. The reintroduction of the HAdV vaccine led to a 300-fold decline in disease burden [22], but this vaccine is not available to the general population. Effective therapeutics are still required to combat HAdV-induced disease, especially in cases of severe, disseminated infections. Several small molecules have shown promise in early preclinical studies and clinical trials, and the development of novel reporter viruses and platforms to screen large drug libraries will allow for the identification of new anti-HAdV compounds and therapeutic targets. Such drugs, upon further development, may have significant benefits in ameliorating HAdV-associated illnesses, and help prevent the spread of the virus in both the general and high-risk populations.

## Figures and Tables

**Table 1:** Novel small molecules with anti-HAdV activity discussed in this review.

Category	Compound	Reference(s)
Nucleoside/nucleotide analogues	Zalcitabine	[118,122,123]
	Alvoudine	[118]
	Stavudine	[124]
	Acyclic nucleoside phosphonate analogues of cidofovir	[118]
	Various ether lipid-ester prodrugs of cidofovir	[125]
	Gemcitabine	[126]
	Cytarabine	[127]
	Other nucleoside/nucleotide analogues	[128]
Inhibitors of epigenetic regulators	Valproic acid	[129]
	Vorinostat	[130]
	Trichostatin A	[130]
	Other HDAC inhibitors	[130]
	Chaetocin	[126]
	GSK126	[131]
	GSK343	[131]
	Lestaurtinib	[126]
Steroid-based compounds	Digoxin	[127,132]
	Digitoxin	[132]
	Digitoxigenin	[127]
	Lanatoside C	[127]
	Dexamethasone	[127]
	Flunisolide	[127]
	Mifepristone	[133]
	Epiandrosterone and derivatives	[134]
Other compounds	Piperazine derivatives	[135,136]
	Tazarotene	[137]
	Ivermectin	[138]
	Protease inhibitors	[139,140]
